# Supplementary material for: Clinical and Forensic Aspects of the Management of Child Abuse: The Experience of the Paediatric Emergency Department in Novara, North-West Italy
Source: Int J Environ Res Public Health. 2023 Jan 22;20(3):2028. doi: 10.3390/ijerph20032028 (PMC9916143; doi:10.3390/ijerph20032028)
Supplement: Supplementary file 1 [file ijerph-20-02028-s001.zip › ijerph-2135278-supplementary.pdf]

CLINICAL AND FORENSIC ASPECTS OF THE MANAGEMENT OF CHILD ABUSE: THE EXPERIENCE OF A PEDIATRIC EMERGENCY DEPARTMENT IN NORTH-WEST ITALY

Supplementary Material

Suppl. Mat. S1: Additional Ethical Considerations

About consent

The consent of the interested parties and their legal representatives was not required for the following reasons:

1) the data minimization process, implemented from the moment of collection pursuant to art. 89 GDPR, leads us to believe that the possibility of identifying the subject to whom the data refers can be considered as minimal and completely residual: the data strictly necessary for the purposes of the research are coded at the source, without links capable of tracing them back to the identity of the interested parties.

2) the processing of data is "necessary for the conduct of studies carried out with data previously collected for health care purposes or for the execution of previous research projects", in accordance with the provisions of the Guarantor to the processing of particular categories of data, pursuant to art. 21, paragraph 1 of Legislative Decree 10 August 2018, n. 101 (Register of measures no. 146 of 5 June 2019), Annex 1.5 Requirements relating to the processing of personal data carried out for scientific research purposes (aut. Gen. No. 9/2016).

3) with reference to the Provision (at point 5.3) of the Guarantor, the circumstances for which in this study it was not possible to obtain the consent of the interested parties are declared:

4.1 ethical reasons: it becomes problematic to bring people, minors back to the time of abuse and in many cases now of age, to remember abuses / violence / mistreatment suffered in the past against will; this could lead to an alteration of the psychological balance achieved over the years.

4.2 reasons for organizational impossibility: many of the minors affected by abuse / violence / mistreatment could have changed residence or may have been removed from their family of origin, thus making it difficult to find information about their current family or the host community.

4.3 the inability to contact most of the interested parties makes the research impossible or significantly alters the results, in an area that needs to be monitored to provide useful data for the prevention of the phenomenon and for implementing procedure to take care of the survivors.

Methods of data processing: anonymization process

The data were deprived of identity elements up to their anonymization.

In particular:

- the data were collected only by the staff of the Paediatric SCDU, which had taken care of the minors to whom the data refer.
- to each case, the staff of the SCDU assigned a random alphanumeric code at the origin so that the researchers, who process what was reported in the database, was not able in any way to trace the minor concerned.
- no link was established between the alphanumeric code and the identity of the minors (as occurs in the pseudonymization process), and therefore it will be very problematic to trace the identity of the minor, considering the process of careful data minimization described below in compliance of the art. 89 GDPR:

• name and surname and initials were not collected.

• the date of birth was not collected, but only the age of the minor was indicated.

• the gender (male, female, other) was collected.

• the place of birth was not collected, but only the country was indicated.

• the precise date of entry into the emergency room was not collected, but only the year and the semester were recorded

• the characteristics that allow to identify the place where the abuse took place were not noted, but generic terms such as home, private place, public place were used

• the research did not foresee follow-up.

The collected data were analysed, immediately aggregated, and used by categories / range.

All operations were carried out only by personnel duly trained and authorized: theytook place in compliance with professional and office secrecy, and principles of correctness, lawfulness, and transparency, in accordance with the provisions of current legislation.

Suppl. Mat. S2: Raw data collection sheet

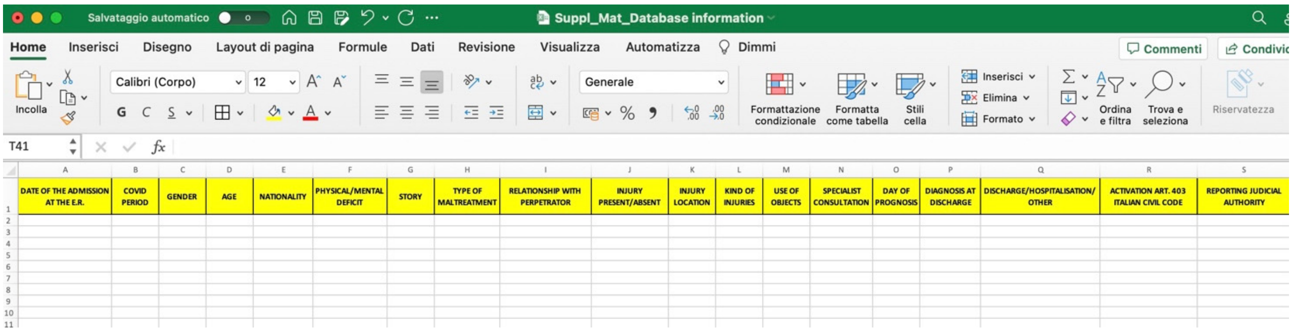

Suppl_Mat_Database information

DATE OF THE ADMISSION AT THE E.R.	COVID PERIOD	GENDER	AGE	NATIONALITY	PHYSICAL/MENTAL DEFICIT	STORY	TYPE OF MALTREATMENT	RELATIONSHIP WITH PERPETRATOR	INJURY PRESENT/ABSENT	INJURY LOCATION	KIND OF INJURIES	USE OF OBJECTS	SPECIALIST CONSULTATION	DAY OF PROGNOSIS	DIAGNOSIS AT DISCHARGE	DISCHARGE/HOSPITALISATION/ OTHER	ACTIVATION ART. 403 ITALIAN CIVIL CODE	REPORTING JUDICIAL AUTHORITY
